# Management of Depression in Chronic Care Patients Using a Task-Sharing Approach in a Real-World Primary Health Care Setting in South Africa: Outcomes of a Cohort Study

**DOI:** 10.1007/s10597-023-01108-y

**Published:** 2023-03-24

**Authors:** Tasneem Kathree, Max Bachmann, Arvin Bhana, Merridy Grant, Ntokozo Mntambo, Sithabisile Gigaba, C. G. Kemp, Deepa Rao, Inge Petersen

**Affiliations:** 1 School of Nursing and Public Health, University of KwaZulu-Natal, Howard College, Mazisi Kunene Road, Durban, 4001 South Africa; 2 School of Applied Human Sciences, University of KwaZulu-Natal, Howard College, Mazisi Kunene Road, Durban, 4001 South Africa; 3 Norwich Medical School, University of East Anglia, Norwich, Norfolk, UK; 4 Health Systems Research Unit, South African Medical Research Council, 491 Peter Mokaba Ridge Road, Overport, Durban, South Africa; 5 Department of international health, Johns Hopkins University, Baltimore, MD USA; 6 Department of Global Health, Department of Psychiatry and Behavioral Sciences, University of Washington, Seattle, United States of America

**Keywords:** Depression, Integrated collaborative care, Chronic disorders, Primary health, Implementation science, Evaluation, Scale-up, South Africa

## Abstract

Depressive symptoms are common in South African primary care patients with chronic medical conditions, but are usually unrecognised and untreated. This study evaluated an integrated, task-sharing collaborative approach to management of depression comorbid with chronic diseases in primary health care (PHC) patients in a real-world setting. Existing HIV clinic counsellors provided a manualised depression counselling intervention with stepped-up referral pathways to PHC doctors for initiation of anti-depressant medication and/ or referral to specialist mental health services. Using a comparative group cohort design, adult PHC patients in 10 PHC facilities were screened with the Patient Health Questionnaire-9 with those scoring above the validated cut-off enrolled. PHC nurses independently assessed, diagnosed and referred patients. Referral for treatment was independently associated with substantial improvements in depression symptoms three months later. The study confirms the viability of task-shared stepped-up collaborative care for depression treatment using co-located counselling in underserved real-world PHC settings.

**Supplementary Information:**

The online version contains supplementary material available at 10.1007/s10597-023-01108-y.

## Introduction

The South African health care system is confronted with a substantial burden of untreated mental illness, including depression. The lifetime prevalence of common mental disorders (CMDs) in South Africa is estimated at 30%, with major depression at 9.8% (Herman et al., 2009). The public mental health burden in South Africa is compounded by a constellation of factors that include a rise in non-communicable diseases (NCDs), multimorbidity in all age groups, which is likely propelled by HIV and hypertension including in younger adults (Roomaney et al., 2022), and the clustering of common risk factors for NCDs in people with mental health conditions (Stein et al., 2019). Co-morbidity of depression in people with NCDs in South Africa is common, with one study in the Western Cape indicating that almost half of participants (45%) with hypertension, diabetes or respiratory disease had depression (Folb et al., 2015). Common mental disorders (CMD) in people with HIV is also reported to be high, ranging from 14 to 34.9% (Freeman et al., 2008; Myer et al., 2008; Olley, Seedat, & Stein, 2006). KwaZulu-Natal is home to the highest burden of HIV in the country (HSRC, 2017), which is often comorbid with hypertension (Roomaney et al., 2022 ; with depression also common, but undetected because of limited investments in mental health (Docrat et al., 2019). Based on the mental health spend for public health care in South Africa, with an estimated 84% of the public reliant on public health services for health care, the treatment gap for mental and neurological disorders is estimated at over 90% (Docrat, Besada, Cleary, Daviaud, & Lund, 2019). Moreover, challenging health care needs and inferior outcomes associated with patients with multiple morbidities amplify the burden on the health care system in providing effective care to patients with comorbid CMDs, including depression (Ivbijaro et al., 2014).

Patient rights to mental health care and guidelines for the provision of mental health care were advanced through the Mental Health Care Act 2002 (Government Gazette, 2002). Building on this foundation, the National Mental Health Policy Framework and Strategic Plan 2013–2020 (South African National Department of Health, 2013) provided a strategy to address the mental health service gap by recommending task-sharing at a primary health care (PHC) level to promote access to mental health services in the population. Task-sharing is based on the principle of devolving tasks among health teams from higher to lower-skilled personnel to address the shortage of health workers and promote efficient use of human resources, to address unmet mental health care needs in scarce resource contexts (Hoeft et al., 2018). Task-sharing of health care requires planning, ongoing training, and support and supervision for different cadres of health workers in the resource mix. However, the feasibility of task-sharing hinges on the successful integration of mental health care into general health services (Spedding, 2015). Models that advocate integrated care by single providers for patients presenting with multiple chronic disease conditions comorbid with mental health conditions may sometimes be unsuitable, particularly where complex assessment and treatment options are required (Ee et al., 2020).

In contrast, integrated collaborative care models for depression comprise multidisciplinary teams of health providers who provide packages of care for physical and mental health conditions to PHC patients. Case managers typically coordinate care and monitor progress (Katon, Unützer, Wells, & Jones, 2010). In the context of a lack of mental health specialists, particularly in low-middle-income-countries (LMIC) collaborative care may include task-sharing components (Acharya et al., 2017). Integrated task-sharing collaborative care models for depression have been successfully implemented in high income countries (Ivbijaro et al., 2014; Katon, 1999), and while showing promise (Ali et al., 2020; Ngo et al., 2014; Petersen, Fairall, et al., 2021), there is a need for more evidence-based collaborative care models for depression in LMIC (Cubillos et al., 2020; Spedding, 2015; van Ginneken et al., 2013).

In South Africa, the PRogramme for Improving Mental health CarE (PRIME), a multinational research consortium supporting the development, implementation and scale-up of integrated mental health care at PHC level in five LMIC (Lund et al., 2012), co-developed and evaluated a collaborative, integrated, task-shared model for depression in patients with chronic diseases comorbid with depression at PHC level in collaboration with the South African Department of Health. In this model, the intervention focussed on capacitating PHC professional nurses to identify chronic care patients with depression through strengthened training in the use of mhGAP guidelines integrated into the chronic care guidelines used in South Africa, called Adult Primary Care (APC) (National Department of Health, 2019/2020). Referral pathways were strengthened through introducing project-employed facility-based lay counsellors trained and supervised to provide a manualised counselling intervention. Referral pathways were stepped-up to existing services provided by consulting PHC doctors for initiation of anti-depressant treatment and/or mental health specialist services at a district hospital level.This was evaluated through an initial cohort study (Petersen et al., 2019) followed by a parallel cluster randomised controlled trial in the North West province in South Africa using project employed lay counsellors that demonstrated non-inferiority to usual care and where referral pathways were only to doctors and specialists (Petersen et al., 2021).

Scaffolding off the PRIME model, the Southern African Research Consortium for Mental health INTegration (SMhINT) project used implementation science to iteratively evaluate and strengthen the implementation of this model in a real-world PHC setting (Petersen et al., 2021; Petersen, van Rensburg, Gigaba, Luvuno, & Fairall, 2020), using lay counsellors in the employ of the KwaZulu-Natal Department of Health to provide the facility-based counselling service.

The aim of this study was to evaluate the viability of the real-world implementation of this collaborative care model on depression symptom reduction in chronic PHC care patients based on nurse diagnosis and referral. More specifically we sought to determine whether patients who were diagnosed with depression and referred for counselling by existing facility-based HIV counsellors in the system, reported improved depression outcomes compared to those who were diagnosed with depression and not referred, and those who were not diagnosed with depression.

## Methods

### Setting

The study was conducted in one of three health sub-districts in the Amajuba district in the KwaZulu-Natal province, South Africa. The district population at the time of the study was estimated to be 530 477, with a density of 76.8 people per km^2^ (COGTA, ND). Over 90% of the population was uninsured for medical cover, and dependant on either out of pocket or public health care (Health Systems Trust, 2016). The study was conducted in the largest and most populous sub-district with a population of 363 236 people served by 14 PHC clinics. Four clinics were excluded for various reasons, including accessibility for research staff due to the remoteness of locations.

### Sample

Adult patients aged 18 and above attending chronic care services in 10 clinics who scored ≥ 9 on the Patient Health Questionnaire-9 (PHQ-9), who did not have intellectual disability and could provide informed consent, and were not at the clinic on the day specifically seeking care for an acute medical issue.

### Study Procedure

Patients in the waiting rooms of the 10 PHC clinics were informed about the study, and volunteers were invited to participate. Trained fieldworkers screened patients using the PHQ-9 and enrolled patients who scored ≥ 9 into the study following informed consent procedures. This cut off was based on a previous validation study of the PHQ-9 in a chronic care population in South Africa (Bhana et al., 2019), Nurses were blinded to the PHQ-9 score and independently assessed the patient using the nationally adopted APC guideline for mental health assessment for use by primary health care (PHC) nurse clinicians, which includes questions on low mood and difficulty in coping, in the mental health assessment. APC provides a prompt for nurses to assess patients who answer affirmatively to either question on the core features of depression using 2 questions from the Whooley screening (Bosanquet et al., 2015) (based on PHQ-2 questions but over past month rather than 2 weeks, and with yes/no responses rather than a scoring system), and provides guidelines on the management of depression including referral for further treatment (Pers.com). The nurses completed a research checklist independently of the PHQ-9 scores to indicate whether they had made a diagnosis of depression and whether they had referred diagnosed patients for further care and treatment. Based on the nurse assessment, participants were categorised into three groups: patients with a diagnosis of depression and referred for treatment, patients with a diagnosis of depression and not referred for treatment and patients without a diagnosis of depression. Between April and September a total 627 participants were recruited. Of the total recruited, 411 were recruited into the study prior to consultation with the nurse. In an effort to increase the power and precision of camparisons between the groups as well as to ensure an adequate number of patients diagnosed and referred for treatment (Kemp et al., 2020), the remaining 216 participants recruited after their consultation with a nurse were included in the analysis, as nurses completed the nurse checklist independently and blind to these patients’ PHQ-9 scores. The survey questionnaires were administered at baseline and at 3 and 9 months after baseline in private spaces at the clinic facilities in either isiZulu, the predominant local language, or in English using handheld tablets.

### Measures

The survey questionnaire included measures of severity of depression symptoms – the primary outcome, and participant characteristics potentially associated with both depression symptoms and the management of depression. Measurement and statistical adjustment for these potentially confounding variables was intended to reduce bias in estimation of effects of diagnosis and referral on depression outcomes. Questions on participants’ socidemographic characteristics including sex, highest educational level attained, monthly household employment and welfare grant income, economic activity (employed or unemployed), household hunger, previous health service utilisation, and previous diagnosis of depression, HIV, asthma, chronic obstructive pulmonary disease, hypertension, heart disease, stroke, tuberculosis, diabetes, high cholesterol, arthritis, epilepsy, and other chronic and mental disorders.

The questionnaire included several health measurement scales. The 9-item Patient Health Questionnaire (PHQ-9) was used to screen for depression and evaluate changes in depression outcomes (Bhana et al., 2019; Bhana, Rathod, Selohilwe, Kathree, & Petersen, 2015; Kroenke, Spitzer, Williams, & Lowe, 2010). The 10-item Alcohol Use Disorders Identification Test (AUDIT) (Saunders et al., 1993) assessed severity of alcohol use disorders. The Perceived Stress Scale (PSS − 10 items) (Cohen et al., 1983) assessed coping and stress in the past month. The World Health Organization Disability Scale (15 items) assessed disability and difficulty coping physically (WHODAS) (Garin et al., 2010). A 3-item Oslo scale assessed social support and social relationships (Dalgard et al., 2006). The Patient Assessment of Care for Chronic Conditions (PACIC) comprised 20 items which assessed patient experiences of care including consultation and advice/education from case managers regarding the management of their conditions (Glasgow et al., 2005). All of the scales were Likert scales, with responses to each item coded as four or five ordered integers, indicating strength or frequency of response, which then were added to produce the relevant scale. All of these scales have previously found to be valid in South Africa (Myer et al., 2008; Garin et al., 2010; Vythilingum et al., 2012; Bhana et al., 2015).

### Description of the Intervention

The intervention was modelled on the PRIME collaborative care package for depression trialled in the North West province in South Africa (Petersen et al., 2022) and included elements of health systems strengthening at district and PHC levels. The Integrated Clinical Services Management health systems strengthening package introduced by the National Department of Health in South Africa (Mahomed & Asmall, 2015; Mahomed, Asmall, & Freeman, 2014) provided the opportunity to expand on this health systems strengthening initiative for the identification and management of care for depression in PHC. The description of the health systems strengthening for depression care follows. In this model, nurses and lay counsellors were trained in aspects of depression management.

#### Nurses

Professional PHC nurses were trained to identify and manage adult patients with communicable diseases, NCDs, women’s health and mental illnesses using the APC clinical decision support tool (Cornick et al., 2018). Training was cascaded via district-based Master trainers and facility-based trainers to capacitate professional nurses to identify depression, provide psychoeducation and refer patients to co-located lay counsellors and /or PHC doctors for initiation of antidepressant treatment or to mental health specialists.

#### Lay Counsellors

Referral pathways for the identification and management of depression were further strengthened to include co-located, lay-counsellor delivered counselling in PHC facilities. Given the human resource constraints for mental health at PHC and long wait times to access specialist mental health care (Petersen et al., 2022), HIV counsellors were trained to deliver a manualized counselling intervention for patients with depression which provided specific guidelines for the administration of the counselling programme contained in a manual for ease of training and reference, and to promote consistency and fidelity to the programme throughout its use in any setting. The intervention was previously tested using project-employed lay counsellors in the PRIME and CobALT trials (Petersen et al., 2019; Petersen, Fairall, et al., 2021; Selohilwe, Bhana, Garman, & Petersen, 2019). In the current study, HIV counsellors were trained by a project-led team of trainers including a clinical psychologist, a registered psychological counsellor, and an adult education specialist. Counsellors received a five-day training off-site, and were supervised by the psychological counsellor. The lay counsellors used a fidelity checklist for self-assessment which was compared against an assessment completed by the registered psychological counsellor. The fidelity checklist assessed whether the lay counsellor followed the structure of the session as trained, applied the basic counselling skills taught, and conducted a risk assessment and/or referred patients who verbalized suicidal ideation for an assessment by the nurse. Each lay-counsellor received a minimum of 3 in-vivo supervision sessions to provide the supervisor with an opportunity to assess the counsellor’s competency in teaching patients 3 main skills: (a) psychoeducation on depression and its causes, (b) problem-solving, and (c) behavioural activation and challenging negative thoughts. Additional support was provided by the psychological counsellor if required following the in-vivo supervision. The psychological counsellor provided ongoing supervision and emotional support in a group setting where lay counsellors submitted their statistics and reported any challenges related to providing the intervention.

Group-based and individual counselling sessions were offered in isiZulu and English to accommodate the predominantly isiZulu-speaking population although only individual sessions were taken up due to challenges including limited availability of counsellors, difficulty in appointing the minimum 5 patients to attend the group at times that were convenient for patients, and lack of appropriate spaces for group sessions. The counsellors also delivered educational talks in the waiting rooms to encourage help-seeking for depression and to raise awareness on treatment options available. The counselling intervention comprised 8 sessions including an introductory psychoeducational session on depression, and used vignettes, and cognitive behavioural techniques to manage the common triggers of depression identified in previous studies (Petersen et al., 2019). These were poverty, interpersonal conflict, social isolation, grief and bereavement, internalized and externalized stigma and a closure session. An additional session on “Getting to know your chronic conditions and medication” was added to encourage adherence to medication.

#### Research Design

A comparison group cohort design was adopted. Adults with at least one chronic medical condition who screened positive for depressive symptoms on the PHQ-9 administered by the fieldworkers were enrolled into the study, and followed up at 3 and 9 months. Changes in depression outcomes were compared at 3 and 9 months among three groups assessed by the PHC nurse, i.e., those not diagnosed with depression, those diagnosed with depression but not referred for treatment, and those diagnosed with depression and referred for treatment.

#### Ethical Considerations

The purpose of the study was initially verbally explained to potential participants by fieldworkers. Upon verbal consent, the study details were explained, and questions from the participants were addressed. Upon agreement to continue with the study, participants provided informed signed consent. Participants were given a copy of the information document for their records. The study was performed in accordance with the ethical standards contained in the 1964 Declaration of Helsinki. Ethical approval was obtained from the Biomedical Research Ethics Committee at the University of KwaZulu-Natal (BF190/17) and the National Health Research Database.

### Statistical Analysis

The statistical analysis aimed to (i) identify participant characteristics independently associated with diagnosis and referral for treatment of depression and (ii) estimate independent effects of diagnosis and referral on depressive symptoms after adjustment for relevant baseline characteristics.

To identify participant characteristics independently associated with diagnosis and referral for treatment of depression, participants were divided into three exposure groups: undiagnosed depression; depression diagnosed but not referred; and depression diagnosed and referred. We first compared the characteristics of each group measured at baseline: age, sex, education, employment, household income, household hunger, previous attendance of other clinics, previously diagnosed HIV, hypertension, diabetes, depression or other conditions, PHQ-9 score, PSS, PACIC, OSLO, and AUDIT scores. Each of these variables was compared for the three exposure groups, using logistic, ordinal logistic or linear regression models for binary, ordinal and continuous variables, respectively. To identify baseline characteristics associated with being in each exposure group, we constructed a multinomial logistic regression model with the three exposure groups as outcomes, with diagnosed but not referred as the base outcome, and with all baseline variables as covariates.

To estimate independent effects of diagnosis and referral on depressive symptoms, we compared the following depression symptom outcomes between pairs of the three groups: mean PHQ-9 score at three months and nine months, with linear regression models, and a clinically significant decrease of > 50% in PHQ-9 score (Kroenke et al., 2001) from baseline to three months, and from baseline to nine months, with logistic regression models. We estimated the independent effect on each outcome of being undiagnosed or diagnosed and referred, compared with being diagnosed but not referred after adjustment for baseline characteristics. We used two regression models for each outcome – the first full model included all baseline characteristics as covariates and the second restricted model included only covariates independently associated with the outcome at the 20% significance level. The latter 20% significance level was used, instead of the conventional 5% level, to avoid excluding potential confounders from the more restricted model.

To assess the robustness of the results obtained using the methods described above, we carried out a sensitivity analysis, using propensity score weighting to adjust for baseline differences between patients who were diagnosed but not referred and patients who were diagnosed and referred. Undiagnosed patients were excluded from this analysis to enable estimation of predicted probabilities of referral among diagnosed patients, using a logistic regression model equivalent to that shown in the lower half of Table 1;Linear and logistic regression models were then weighted by the inverse probability of referral, with the binary variable indicating referred versus not referred 1and baseline PHQ-9 score as covariates.

**Table 1:** Baseline characteristics of participants

	Undiagnosed (N = 265)		Diagnosed but not referred (N = 143)			Diagnosed and referred (N = 219)				Total (n = 627)	
	N	%	N	%	P	N	%	P	P*	N	%
Variable	265	100	143	100		219	100			627	100
Sex (female)	203	76.6	110	76.9	0.98	170	77.6	0.86	0.91	483	77.0
Education (completed secondary school)	59	22.3	31	21.7	0.78	51	23.2	0.83	0.72	141	22.5
Employed	88	33.2	41	28.7	0.43	58	26.5	0.20	0.24	187	29.8
Low salary (< R2000pm)	234	88.3	135	94.4	0.04	205	93.6	0.02	0.66	574	91.6
Household hunger	104	39.4	66	46.2	0.12	106	48.4	0.07	0.70	276	44.1
Visited another clinic in last 3 months	34	12.8	17	11.9	0.80	24	11.0	0.65	0.47	75	12.0
HIV	200	76.1	111	77.6	0.75	183	83.6	0.12	0.46	494	79.0
Hypertension	17	27.0	41	28.7	0.73	58	26.5	0.93	0.77	170	27.2
Diabetes	20	7.6	15	10.5	0.22	12	5.5	0.16	0.08	47	7.5
Other chronic condition/s	68	25.7	37	25.9	0.96	46	21.0	0.16	0.38	151	24.1
Previous diagnosis depression	8	3.0	7	4.9	0.25	27	12.3	0.01	0.22	42	6.7
PHQ-9 score category					0.001			0.005	0.66		
Mild/moderate (6–14)	220	83.0	84	59.2		138	63.0			442	70.6
Moderately severe (15–19)	35	13.2	41	28.9		58	26.5			134	21.4
Severe (20–27)	10	3.8	17	12.0		23	10.5			50	8.0
Recruited after nurse consultation	43	16.2	22	16.1	0.98	150	68.5	0.001	0.001	215	34
	mean	SD	mean	SD	P	mean	SD	P	P*	mean	SD
Age (years)	44.1	13.6	44.2	12.7	0.94	43.2	13.5	0.65	0.47	43.8	13.4
PACIC score	8.3	8.8	7.6	8.9	0.54	7.3	8.8	0.40	0.75	7.8	8.8
PSS score	18.3	5.0	19.5	5.9	0.17	18.0	5.4	0.73	0.27	18.4	5.4
Oslo score	9.1	2.4	8.6	2.7	0.22	8.3	2.5	0.02	0.27	8.7	2.5
WHODAS score	21.7	8.1	24.9	8.6	0.001	24.1	9.5	0.26	0.31	23.2	8.8
PHQ-9 score	10.8	4.2	14.0	4.5	0.001	13.5	13.5	0.01	0.49	12.5	4.7
AUDIT score	5.0	8.6	5.0	6.5	0.99	3.4	3.4	0.03	0.28	4.4	7.1
Number of chronic conditions (median/IQR)	1	1–2	1	1–2	0.47	1	1	0.12	0.67	1	1–2

All P values for comparison with undiagnosed group, except P* value for comparison with diagnosed unreferred group. All P values adjusted for intra-clinic correlation, with logistic regression for binary variables, original logistic regression for PHQ-9 categories and number of chronic conditions, and linear regression for continuous variables. SD standard deviation. IQR interquartile range

All regression models accounted for intra-cluster correlation of outcomes among clinics with Huber-White robust adjustment. All multivariable regression models included a covariate indicating whether participants were recruited before or after the change in enrolment method. We used a two-tailed 5% significance level. All statistical analyses used Stata 17 software (StataCorp, 2021).

All authors certify responsibility for the methods and analysis.

## Results

### Characteristics of Participants at Baseline

Participants’ characteristics at enrolment in the study (baseline) are reported in Table 2.. A total of 627 patients participated in the study, of whom 77% were female, 7% had previously been diagnosed with depression, 79% diagnosed with HIV, and 27% had a diagnosis of hypertension. Only 30% were employed, 23% had completed secondary school education, and 44% reported experiencing hunger in their households in the past month. Participants’ baseline PHQ-9 scores indicated 71% with mild or moderate depression, 21% with moderately severe depression and 8% with severe depression.

**Table 2:** Predictors of diagnosis and referral: multinomial logistic regression model

Outcome: Undiagnosed vs Diagnosed but not referred	Relative risk ratio	Lower 95% confidence limit	Upper 95% confidence limit	*p*
Sex (female)	1.55	0.88	2.74	0.13
Education (matric)	1.05	0.63	1.76	0.84
Employed	0.77	0.38	1.57	0.48
Low salary (< R2000pm)	0.42	0.16	1.08	0.07
Household hunger	0.72	0.47	1.09	0.12
Visited another clinic in last 3 months	1.13	0.51	2.53	0.76
HIV	1.24	0.48	3.22	0.66
Previous diagnosis depression	0.96	0.20	4.74	0.96
Hypertension	1.35	0.23	7.98	0.74
Diabetes	1.29	0.46	3.63	0.62
Other chronic condition/s	2.02	0.73	5.55	0.17
Number of chronic conditions	0.67	0.27	1.68	0.39
PHQ-9 score	0.85	0.78	0.92	< 0.001
Age (years)	1.00	0.98	1.03	0.74
PACIC score	1.02	0.99	1.06	0.13
PSS score	1.01	0.96	1.05	0.82
Oslo score	1.03	0.93	1.14	0.56
WHODAS score	0.98	0.96	1.01	0.18
	0.99	0.95	1.04	0.72
AUDIT score Recruited after nurse consultation	0.98	0.43	2.22	0.97

Outcome: Undiagnosed vs Diagnosed but not referred	Relative risk ratio	Lower 95% confidence limit	Upper 95% confidence limit	*p*
Sex (female)	1.23	0.61	2.49	0.57
Education (matric)	1.33	0.67	2.62	0.42
Employed	0.85	0.52	1.38	0.50
Low salary (< R2000pm)	0.86	0.41	1.80	0.69
Household hunger	1.14	0.71	1.85	0.58
Visited another clinic in last 3 months	0.97	0.54	1.74	0.91
HIV	2.46	0.72	8.41	0.15
Previous diagnosis of depression	7.48	1.45	38.49	0.02
Hypertension	2.96	0.66	13.37	0.16
Diabetes	0.97	0.28	3.29	0.96
Other chronic condition/s	1.36	0.60	3.08	0.46
Number of chronic conditions	0.65	0.35	1.22	0.18
PHQ-9 score	0.99	0.94	1.04	0.64
Age (years)	1.00	0.98	1.03	0.75
PACIC score	1.01	0.98	1.05	0.42
PSS score	0.93	0.88	0.99	0.03
Oslo score	0.96	0.88	1.04	0.27
WHODAS score	1.02	0.97	1.06	0.48
AUDIT score	0.99	0.95	1.04	0.75
Recruited after nurse consultation	12.88	5.44	30.50	< 0.001

*p* values and confidence intervals adjusted for intra-cluster correlation of outcomes

### Diagnosis and Referral of Participants and Baseline Differences Between Those Undiagnosed with Depression, Diagnosed with Depression and Not Referred for Treatment, and Diagnosed and Referred for Treatment for Depression

Depression was not diagnosed by a nurse in 265 (42%) patients during the consultation; 143 (23%) were diagnosed with depression but were not referred for treatment for depression, and 219 (35%) were diagnosed with depression and referred for treatment. Patients were referred mainly to the co-located counsellor, with just three (0.5%) patients also referred to a psychologist, and no referrals made to a PHC doctor. Compared with undiagnosed patients, those diagnosed but not referred were significantly more likely to report low salaries and higher PHQ-9, WHODAS and OSLO scores, and diagnosed and referred patients were significantly more likely to report low salaries and higher PHQ-9, OSLO and AUDIT scores (Table 2). There were no significant differences between diagnosed but not referred patients and diagnosed and referred patients.

Participant characteristics independently associated with diagnosis and referral for treatment of depression are shown in Table 3. In the multivariable model, having a previous diagnosis of depression, a lower PSS score, and recruitment after the nurse consultation were statistically significantly associated with being diagnosed and referred, compared to being diagnosed but not referred. Having a lower baseline PHQ-9 score was statistically significantly associated with being undiagnosed, compared to being diagnosed but not referred.

**Table 3:** PHQ9 scores and changes in scores at baseline and follow up

	Undiagnosed		Diagnosed but not referred			Diagnosed and referred				Total	
	mean	SD	mean	SD	*p*	mean	SD	*p*	p*	N	%
Baseline	(n = 265)		(n = 143)			(n = 219)				(n = 627)	
	10.8	4.2	14.0	4.5	0.001	13.5	4.5	0.007	0.49	12.4	4.7
3 months	(n = 181)		(n = 114)			(n = 151)				(n = 446)	
	6.4	4.7	7.8	5.7	0.19	5.5	4.5	0.05	0.05	6.4	5.0
9 months	(n = 175)		(n = 98)			(n = 142)				(n = 415)	
	5.2	4.5	6.6	5.9	0.39	5.0	5.0	0.28	0.28	5.4	5.0
Change baseline to 3 months	– 4.6	6.1	−6.3	6.4	0.16	– 8.1	6.4	0.02	0.02	– 6.2	6.5
Change baseline to 9 months	– 5.8	6.1	–7.5	6.6	0.27	– 8.6	6.3	0.05	0.25	– 7.1	6.4
> 50% reduction:	n/N	%	n/N	%	*p*	n/N	%	*p*	p*	N	%
• Baseline to 3 months	86/181	47.5	57/114	50.0	0.73	100/151	66.2	0.03	0.07	243/446	54.5
• Baseline to 9 months	97/175	55.4	52/98	53.0	0.82	89/142	62.7	0.32	0.32	238/415	57.4

All P values for comparison with undiagnosed group, except p* value for comparison with diagnosed unreferred group. All *p* values adjusted for intra-clinic correlation, with logistic regression for binary variables, and linear regression for continuous variables. SD standard deviation

## Changes in Depression Outcomes, and Effects of Diagnosis and Referral on Depression Outcomes

Follow-up rates were similar in the three groups (Table 1). Of 627 participants interviewed at baseline, 446 (71%) were followed up at three months, and 415 (66%) were followed up at nine months. Of 265 participants in whom depression was not diagnosed by a clinic nurse, 181 (68%) and 175 (66%) were followed up at three and nine months, respectively. Of 143 participants in whom depression was diagnosed by a clinic nurse but who were not referred for treatment of depression, 114 (80%) and 98 (69%) were followed up at three and nine months, respectively. Of 219 diagnosed and referred participants, 151 (69%) and 142 (65%) were followed up at three and nine months, respectively.

At baseline, mean PHQ-9 scores were significantly higher in both diagnosed and not referred and diagnosed and referred patients compared to undiagnosed patients but did not differ between the two diagnosed groups (Table 1). Three months later, mean PHQ-9 scores were significantly lower in diagnosed and referred patients than in the other two groups. Similarly, the mean decrease in PHQ-9 scores at three months was significantly greater in diagnosed and referred patients than in the other groups (mean = 4.5 vs. 7.8 and 6.4). The percentage of patients with more than 50% reduction in PHQ-9 from baseline to three months was significantly higher in the diagnosed and referred group than in the undiagnosed group (66.2% vs. 47.5%) but did not differ significantly from the diagnosed but not referred group (50.0%). At nine months follow-up there were no significant differences between the three groups in mean PHQ-9 score, change in score from baseline, or percentage with greater than 50% reduction in PHQ-9 from baseline.

The estimated independent effects of diagnosis and referral on depressive symptoms after adjustment for relevant baseline characteristics are shown in Table 4. Compared to patients who were diagnosed but not referred, diagnosed and referred patients had independently and significantly lower mean PHQ-9 scores at three months (-1.79 difference in means), and were significantly more likely to have decreased PHQ-9 scores by more than 50% from baseline to three months (odds ratio 2.07) in multivariable models with restricted covariates (Table 4). These differences were slightly smaller and not statistically significant in full models that included all covariates. Both outcomes did not differ significantly between undiagnosed patients and patients who were diagnosed but not referred in any model. In equivalent models with PHQ-9 score at nine months and greater than 50% reduction from baseline to nine months as outcomes there was no independently significant difference between the three comparison groups.

**Table 4:** Association between diagnosis and referral with PQ9 score and change in score at 3 months: logistic and linear regression models (n = 418)

	Outcome: PHQ9 score (linear regression models)									Outcome: PHQ3 score decreased > 50% from baseline (logistic regression models)													
	Full model				Restricted model					Full model							Restricted model						
Explanatory variables	Coeff-icient	95% confidence interval		*p*	Coeff-icient	95% confidence interval		*p*		Odds ratio		95% confidence interval			*p*		Odds ratio		95% confidence interval			*p*	
Comparison group:																							
Diagnosed but not referred (reference)																							
Diagnosed and referred	− 1.55	− 3.28	0.17	0.07	− 1.79	− 3.33	− 0.25		0.03		1.79		0.95	3.37		0.07		2.07		1.12	3.85		0.02
Undiagnosed	− 0.69	− 1.91	0.54	0.24	− 0.63	− 2.18	0.92		0.38		1.25		0.90	1.74		0.19		1.22		0.86	1.73		0.27
Sex (female)	0.19	− 1.09	1.48	0.74							0.84		0.58	1.22		0.36							
Education (matric)	− 0.26	− 1.84	1.32	0.72							0.94		0.52	1.73		0.85							
Employed	1.62	0.12	3.13	0.04	1.68	0.21	3.15		0.03		0.56		0.34	0.93		0.03		0.52		0.31	0.85		0.01
Low salary (< R2000pm)	1.65	− 0.86	4.15	0.17	1.83	− 0.74	4.40		0.14		0.54		0.22	1.32		0.18		0.46		0.18	1.21		0.12
Household hunger	1.31	0.22	2.40	0.02	1.26	0.27	2.25		0.02		0.63		0.34	1.17		0.15		0.63		0.34	1.18		0.15
Visited another clinic in last 3 months	0.90	− 0.87	2.67	0.28							0.76		0.41	1.43		0.40							
HIV	− 0.20	− 1.32	0.92	0.69							1.28		0.71	2.28		0.41							
Previous diagnosis of depression	1.08	− 1.13	3.28	0.30							0.41		0.13	1.33		0.14		0.30		0.11	0.81		0.02
Hypertension	− 1.66	− 3.86	0.55	0.12	-1.79	− 3.24	− 0.34		0.02		2.96		0.86	10.15		0.08		1.53		0.80	2.90		0.20
Diabetes	− 1.52	− 3.70	0.66	0.15	-1.77	− 3.87	0.32		0.09		1.60		0.80	3.19		0.18							
Other chronic condition/s	− 0.70	− 2.83	1.43	0.48	-0.93	− 2.15	0.29		0.12		1.16		0.50	2.72		0.73							
Number of chronic conditions	0.76	− 0.26	1.77	0.13	0.97	0.05	1.88		0.04		0.75		0.46	1.25		0.27							
Age (years)	0.02	− 0.01	0.05	0.17	0.03	− 0.01	0.07		0.16		0.98		0.97	1.00		0.01		0.98		0.97	1.00		0.01
PACIC score	− 0.04	− 0.12	0.04	0.27							1.00		0.96	1.03		0.95							
PSS score	0.16	− 0.05	0.37	0.11	0.17	− 0.01	0.35		0.07		0.95		0.89	1.01		0.08		0.95		0.91	1.00		0.07
Oslo score	0.15	− 0.14	0.44	0.26							0.95		0.86	1.05		0.30		0.94		0.87	1.02		0.15
WHODAS score	− 0.03	− 0.15	0.09	0.63							1.01		0.96	1.07		0.63							
AUDIT score	0.04	− 0.07	0.14	0.44							0.98		0.95	1.02		0.32							
Recruited after referral	− 0.33	− 1.93	1.26	0.65							1.27		0.71	2.28		0.41							
PHQ9 score	0.14	− 0.19	0.46	0.37	0.10	− 0.24	0.45		0.51		1.08		0.95	1.23		0.22		1.09		0.97	1.22		0.15

All P values and confidence intervals adjusted for intra-clinic correlation of outcomes

The sensitivity analysis using propensity scores produced similar estimates to the primary analyses. The propensity score-adjusted mean difference in PHQ-9 score at 3 months between diagnosed referred and diagnosed unreferred patients was − 1.95, 95%CI [-3.86 -0.04], *p* = 0.04), and the respective odds ratio of > 50% decrease in PHQ-9 from baseline to three months was 2.02, 95%CI [1.10 3.73], *p* = 0.02). There were no significant differences in these outcomes at nine months. The propensity score-adjusted difference in PHQ-9 at nine months was − 1.69, 95%CI [− 4.79 1.42], *p* = 0.25), and the odds ratio of > 50% decrease in PHQ-9 from baseline to nine months was 1.65, 95%CI [0.69 3.95], *p* = 0.25). The kernel density distributions of propensity scores in the diagnosed referred and diagnosed but unreferred patients are shown in Supplementary Fig. 1. This shows that the propensity scores of diagnosed and referred patients tended to be higher than scores of diagnosed but not referred patients, with overlapping distributions except for a small proportion of diagnosed but not referred patients.

## Discussion

Following the testing of the PRIME collaborative care model in the North West province in South Africa (Petersen et al., 2016, 2019; Petersen, Fairall, et al., 2021), the results of this evaluation demonstrate the viability of the scaled-up model in routine services in a different provincial and sub-district setting. Participants who were diagnosed and referred by the nurse clinician for treatment for depression were more likely to have significantly reduced depressive symptoms at 3-months follow-up than participants diagnosed but not referred for treatment for depression. This suggests that referral and treatment for depression within the collaborative care model effectively reduced depressive symptoms. These effects were, however, mitigated over time as the study shows no difference at 9 months Our study used a validated cut-off of ≥ 9, (Bhana et al., 2015) with close to three quarters of the participants in the cohort sample falling within the mild or moderate depression ( scoring 9–14) as previously mentioned. Internationally, untreated depression is estimated to spontaneously remit in a third of people with depression and is more likely in people with mild to moderate depression (Whiteford et al., 2013). The absence of a difference at 9 months is thus probably as a result of spontaneous remission in the mild to moderate cases which dominated the sample. The PRIME cohort study in South Africa, which used the same intervention, but under more controlled conditions, similarly reported a significantly greater reduction in the treatment group at 3-month follow up compared to the control group; but remission in both groups at 12-month follow-up (Petersen et al., 2019). Given spontaneous remission, particularly in people with mild to moderate symptoms of depression, and in the face of limited resources in LMIC, depression treatment algorithms in these contexts should favour people with moderately severe and severe symptoms.

This sample comprised mostly women, similar to other studies reporting the preponderance of female participants in local PHC settings, attributed to various factors including that women are more likely to be on HIV care while HIV-negative men rarely attend PHC services for any treatment. Regardless of HIV status, antenatal and paediatric care, PHC visits are more frequent among women than men (Randera-Rees et al., 2021). HIV and hypertension which are highly prevalent in KwaZulu-Natal were the most prevalent conditions reported, although not necessarily in combination (Conan et al., 2022; Pillay, Pillay, & Pillay, 2021), and HIV was afforded greater attention by the healthcare system as it was a priority condition. Of the three groups we compared (depression undiagnosed, depression diagnosed but not referred for treatment, and depression diagnosed and referred for treatment), both diagnosed groups had higher PHQ-9 scores at baseline, which suggests that nurse clinicians were more likely to detect depression with increased symptom severity. Patients who were diagnosed with depression before this study were also more likely to be diagnosed by the nurse, although not necessarily referred. Differences noted between the diagnosed groups was that disability was significantly greater in the group diagnosed and not referred, whileless support was significantly greater in the referred group.

The predominance of referrals to the counsellor suggests that the presence of a co-located counsellor provided an accessible pathway to care, encouraging nurses to refer to the counsellor with negligible upward referrals to a mental health specialist (3 out of 219 referred). The collaborative care approach includes referral to PHC doctors for initiation of anti-depressants as this was part of usual care. Despite the presence of moderate to severe depression, none of the participants was referred to a doctor, potentially depriving patients who would have benefitted from pharmacological anti-depressant treatment. These results suggest the need for a targeted approach to strengthen referral to doctors for the initiation of anti-depressants. However, given that PHC doctors are not uniformly available in PHC facilities across the country, there is a need to consider authorising PHC nurses to initiate anti-depressant treatment with the necessary guidelines, training and support in these underserved regions Petersen, Fairall, et al., 2021).

The clinically significant reduction in depressive symptoms of patients who were referred for depression care has notable implications for integrated collaborative task-shared care for chronic care patients with depression. The vast majority were referred to the non-specialist clinic counsellors who were existing HIV counsellors supervised by a study-appointed supervisor. Given that HIV counsellors are being phased out in the health system in KwaZulu-Natal, to support sustainability, it is essential at a policy level to identify suitable cadres of healthcare workers to be trained to provide co-located mental health counselling and a cadre to provide the necessary training and supportive supervision and mentorship. At the time of writing, the HIV counsellors were undergoing training to transition to Social Auxiliary Workers (SAWs), with limited counselling for CMD falling within their scope of practice, provided that they received adequate and appropriate supervision and support from social workers. Additionally, the Department of Health had indicated plans to base registered counsellors (B.Psych) at community health centres (Pers. com). This cadre was well placed to provide a referral resource within PHC level. Potentially, both cadres would add considerable value to and strengthen the mental health system.

The SMhINT stepped up collaborative care model introduced various systems strengthening features, principally enhanced mental health training for PHC service providers to promote identification and management for depression in chronic care patients with co-located counselling in a real-world setting using CBT techniques which have been shown to be as effective as pharamacotherapies in the short term and more effective long-term (Cuijpers et al., 2023). However, in our context, the co-located counselling was predicated on the facility-based HIV counsellors who were also tasked with various other duties and who were not dedicated specifically to the counselling programme. As a result, both referrals and counselling services were compromised by competing priorities (Kemp et al., 2021). In comparison, the HOPE trial in South India (Srinivasan et al., 2022) approached collaborative care for depression comorbid with diabetes and cardiovascular disease (CVD) using a multilevel approach including a PHC-based pharmacological intervention linked to a sustained, community-based counselling and healthy lifestyle intervention over 12 months. (Srinivasan et al., 2022). While this model of dedicated collaborative care demonstrates the potential for facility/community linkage and care, it may be somewhat difficult to replicate and sustain in resource-constrained real-world settings where a designated approach may be more feasible. Evidence from a South African study in the Western Cape reports equal support (40% each) for a designated approach to co-located task-shared mental health services delivered by lay counsellors using existing PHC staff trained to provide this service, and a dedicated approach which proposes the reallocation of funding from secondary and tertiary mental health services to PHC level for recruitment of mental health cunsellors to address the gap in co-located counselling for depression (Sorsdahl et al., 2020). The study also reported some support (20%) for hybrid approaches to tailor co-located counselling, The testing and adoption of appropriate models of care for task-shared, co-located mental health services requires careful consideration of the resources and the need to strengthen the specific health system context for successful integration.

The study’s main limitation is its observational design, which may have led to biased estimates of the effectiveness of referrals. That is, patients who were diagnosed and referred for depression may, at baseline, have been systematically different from patients who were diagnosed and not referred for depression, and these factors may have influenced nurses’ decisions to refer them, or affected their subsequent changes in PHQ-9 scores. Even though we measured and adjusted for important baseline characteristics, and although these two groups had similar PHQ-9 scores at baseline, they may nevertheless have differed with regard to unmeasured prognostic characteristics, and errors in our baseline measurements would also have limited our ability to adjust for them statistically. The robustness of the statistical models used to estimate effectiveness was, however, supported by the sensitivity analysis using propensity score adjustment, which generated similar estimates to the primary analyses. The results concerning predictors of diagnosis and referral differ slightly from those reported in our previous paper (Kemp et al., 2020), partly because they are based on the expanded cohort which comprised a larger group of referred patients. Qualitative results reported in Kemp et al. (2021) offer further insights into reasons for not referring patients for depression care. These included inadequate time to thoroughly assess and refer patients due to heavy workloads, limited access to telephones to link patients to care and inadequate mental health human resources to refer patients to. HIV counsellors trained to provide the depression counselling in each clinic, were also not necessarily available at all times for depression care due to absence or competing tasks, which proved a barrier to referrals. Lower exposure to in-service mental health training was also identified as a barrier to referral (Kemp et al., 2021).

## Conclusion

The findings indicate that training PHC health workers to identify, refer and treat depression within a collaborative task-shared model encouraged the use of co-located/ facility-based counselling delivered by non-specialist mental health workers as part of real-world PHC services, helped to promote integrated, accessible mental health services. The benefits include improved mental health, which may have ripple effects on managing other chronic health conditions, including treatment adherence for other chronic conditions. The next step is to review the lessons learned, adapt and refine the implementation model and test across rural, semi-urban and urban sites in the district.

## Supplementary Information

Below is the link to the electronic supplementary material.

Supplementary material 1 (DOCX 22.9 kb)
