# Supplementary material for: Management of Depression in Chronic Care Patients Using a Task-Sharing Approach in a Real-World Primary Health Care Setting in South Africa: Outcomes of a Cohort Study
Source: Community Ment Health J. 2023 Mar 24;59(7):1261–74. doi: 10.1007/s10597-023-01108-y (PMC10447595; doi:10.1007/s10597-023-01108-y)
Supplement: Supplementary file 1 — Supplementary material 1 (DOCX 22.9 kb) [file 10597_2023_1108_MOESM1_ESM.docx]

**Supplementary Figure. Probability distribution of propensity scores**
